# Bone Marrow Mesenchymal Stromal Cells (BMMSCs) Augment Osteointegration of Dental Implants in Type 1 Diabetic Rabbits: An X-Ray Micro-Computed Tomographic Evaluation

**DOI:** 10.3390/medicina56040148

**Published:** 2020-03-25

**Authors:** Nabeeh Abdullah Alqahtani, Harish C. Chandramoorthy, Sharaz Shaik, Jamaluddin Syed, Ramesh Chowdhary, Leoney Antony

**Affiliations:** 1 Department of Periodontics and Community Dentistry, College of Dentistry, King Khalid University, Abha 62529, Saudi Arabia; 2 Centre for Stem Cell Research and Dept. of Microbiology and Clinical Parasitology, College of Medicine, King Khalid University, Abha 62529, Saudi Arabia; ccharishjabali@gmail.com; 3 Department of Prosthetic Dentistry, College of Dentistry, King Khalid University, Abha 62529, Saudi Arabia; sharazshaik@gmail.com; 4 Department of Oral Basic and Clinical Sciences, Faculty of Dentistry, King Abdul Aziz University, Jeddah 21589, Saudi Arabia; drjamalsyed@gmail.com; 5 Department of Prosthodontics, Raja Rajeshwari Dental College and Hospital, Bengaluru 560074, India; drramc@yahoo.com; 6 Rajah Muttiah Dental College and Hospital, Annamalai University, Chidambaram 608002, India; antony.leoney@gmail.com

**Keywords:** osseointegration, stem cells, diabetes mellitus, x-ray micro-computed tomography (micro-ct), dental implants

## Abstract

*Background and objectives*: The study aimed to investigate the effect of bone marrow mesenchymal stromal cells (BMMSCs) on implant-bone osseointegration in type I diabetic New Zealand rabbits. *Materials and methods:* BMMSCs harvested from healthy rabbits were processed and validated for purity and osteocyte differentiability. Mandibular incisors of diabetic and control rabbits were carefully extracted, and the sockets were plugged with collagen sponges. Platelet-rich plasma (PRP) containing osteoinductive BMMSCs, and plain PRP were injected into the collagen sponge of the right and left sockets respectively. Dental implants of 2.6 mm diameter and 10 mm length were inserted into the collagen sponge of both sockets. All the animals were sacrificed six weeks post surgery to evaluate an early stage of osseointegration; the mandibles scanned by X-ray microcomputed tomography (μCT) and subjected to 3D analysis. The μCT parameters of the right implant were paired against that of the left side of each animal and analyzed by paired T-test. *Results:* The preclinical evaluation of the viability and osteocyte differentiation of the BMMSCs were consistent between both the donor samples. The osseointegration of dental implants with stem cell therapy (BMMSCs + PRP + collagen) in normal and diabetic rabbits was significantly higher than that of implants with adjunctive PRP + collagen only (*p* < 0.05). *Conclusion:* Stem Cell therapy with osteoinductive BMMSCs and PRP can offer a novel approach to enhance the osseointegration of dental implants in uncontrolled diabetic patients.

## 1. Introduction

Uncontrolled diabetes mellitus predisposes to infirm osseointegration and implant failures, especially during the healing phase [1,2,3]. Pathophysiology of impaired wound healing in diabetes mellitus involves the vascular, neurological, and immune systems. There have been extensive studies on associated factors of diabetic complications on wound healing [4,5,6,7], but the remedies to overcome the complications are inadequate. Mild to moderate increase in the implant survival rate has been reported by using chlorhexidine mouth rinses, pre-operative antibiotics, and hydroxyapatite -coated implants [8]. Though reports suggest that insulin therapy and control of hyperglycemia can significantly improve survival [9], it is not always consistently achieved by the patients.

Regenerative dentistry has a promising future for many oral disorders, including tooth replacement. It has evolved from biomaterials (e.g., granules, blocks, composites, sponges, membranes, acellular matrices) to the contemporary stem-cell-based applications such as direct cellular grafts, tissue-engineered grafts, and sheets of induced stem cells [10]. With the anticipation of future clinical applications, stem cell banking is already in practice worldwide. While dentists can imagine the future of regenerative dentistry with a completely bio-engineered prosthetic tooth, the present-day application is extensive in bone grafting and implantology [11]. 

Bone marrow, periosteum, adipose tissue, dental pulp, and salivary glands are potential sources of multipotent adult stem cells [12]. Bone-marrow-derived mesenchymal stem cells (BMMSCs) are known to have high osteogenic ability and anti-inflammatory effects on the injured tissue [10,13]. BMMSCs are a good choice for allogenic transplantation owing to their immuno-privileged and immune-suppressive properties [14]. Studies have shown that BMMSC can be an ideal alternative for tissue regeneration and integration [15,16]. The use of platelet-rich plasma (PRP) as a therapeutic modality for the accelerated healing process of bone and tendons has been well documented [17]. In addition, few studies have used platelet-derived growth factors to induce stem cells especially MSCs of varied origin to enhance osteogenesis [18]. The mechanism behind using PRP or platelet-derived growth factor with MSCs is not clearly understood as reports indicate platelet-derived growth factor does not have the same effect on all types of stromal cells [19]. 

Osseointegration was defined as intimate contact of bone with foreign material; contemporary definition regards osseointegration as a new bone formation to shield off a foreign body [20]. The intimate contact of the bone to the foreign material is conventionally analyzed by histomorphometry; however, studies suggest μCT analysis produces comparable results [21]. There are studies in the literature on the enhancement of bone to implant osseointegration in diabetes mellitus [22,23,24], but little about tissue regeneration by stem cells. We performed the study, aiming to evaluate the effect of stem cell therapy on the implant osseointegration in uncontrolled type I diabetic New Zealand rabbits using μCT analysis.

## 2. Materials and Methods

**Scheme 1:**
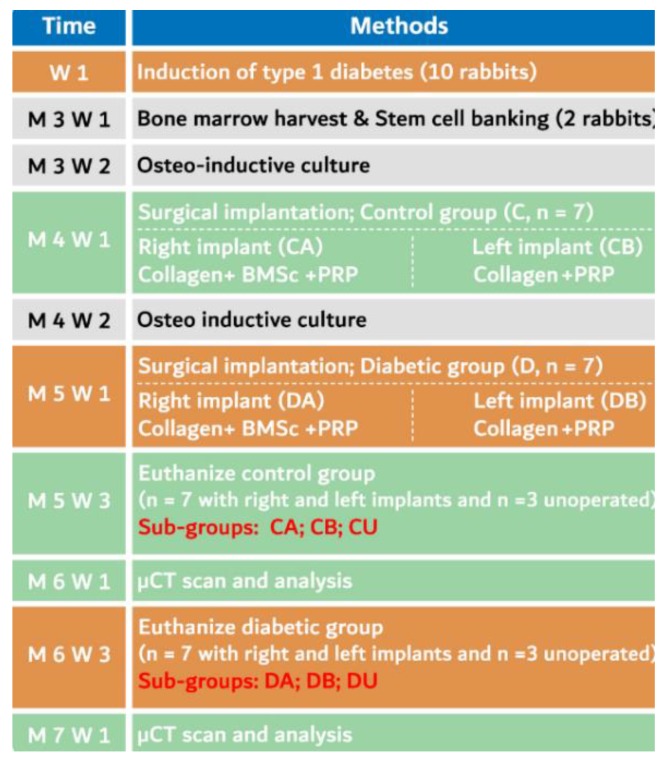
Time-line summary of research design. W: week; M: month; BMSc: bone marrow-derived mesenchymal stem cells; PRP: autologous platelet-rich plasma; μCT: micro CT; C: control, D: diabetic U: unoperated; A: right Implant; B: left Implant; CA: control group with stem cell therapy; CB: control group without stem cell therapy; CU: unoperated control group; DA: diabetic group with stem cell therapy; DB: diabetic group without stem cell therapy; DU: unoperated diabetic group.

The study design and protocol were approved by the research ethics committee of the College of Medicine, King Khalid University vide, letter REC #2018-02-01, dated 19-03-2018. The study included 22 healthy male New Zealand rabbits initially aged around six months and weighing 3.5 kg to 4 kg. During the experiment, two healthy rabbits were sacrificed for BMMSCs and PRP harvesting and the remaining animals were randomly distributed into proportionate groups: control (group C, *n* = 10) and diabetic (group D, *n* = 10). Further subgrouping is illustrated in Scheme 1.

### 2.1. Animal Care

The animals were housed as per the NIH Guideline. Since all the rabbits were mature males, they were individually caged in 3ft×3ft×2ftspace. Wired vertical walls and side-by-side arrangement of cages facilitated olfactory and visual contact between the rabbits. The medication used during the study is tabulated in Table S1 of supplementary material. 

### 2.2. Induction of Diabetes

Alloxan monohydrate (100 mg/kg) in 5 mL normal saline was injected intravenously (i.v.) via marginal ear vein over 2 min (slow i.v. injection) to induce type 1 diabetes in a group of 10 rabbits [25]. During the administration of the alloxan injection, rabbits received an intramuscular (IM) dose of 30 mg/kg ketamine hydrochloride and 3 mg/kg xylazine. Post anesthetic recovery the rabbits were fed with 10% glucose oral solution over a day or two to prevent sudden hypoglycemia. Fasting blood glucose levels were extensively monitored for a week starting from 72 hours with the help of a glucometer (Accu-chek, Roche, Basel, Switzerland) as per manufacturer instructions. Consistent six-hour fasting blood glucose levels of above 300 mg/dL, two weeks post induction was considered as successful induction of diabetes. These rabbits received a second dose of alloxan (100 mg/kg) by i.v. to maintain the 300 mg/dL glucose margin. Fasting blood glucose was monitored once per week to determine the consistency of diabetes. Subcutaneous infusion of dextrose normal saline and insulin therapy was given at appropriate times if the fasting blood glucose levels were more than 350 mg/dL to prevent hyperglycemic shock. The insulin dose was adjusted with the blood glucose level by 1 unit/kg for every 100 mg/dL rise above 350 mg/dL. If the rabbit fasting blood glucose levels reached 600 mg/dL, the second dose of insulin was given in the afternoon. 

### 2.3. Autologous Platelet Rich Plasma (PRP) Preparation 

A pair of healthy rabbits were sacrificed, and whole blood was collected by heart puncture, into sodium citrate tubes. The blood was centrifuged at 180× *g* for 10 min and the supernatant was transferred into a sterile tube for the second round of high speed (890× *g*) centrifugation for 10 min [26]. The sediment, which is rich in platelets (PRP), was collected and resuspended in the minimal plasma until further use.

### 2.4. BMMSCs Sourcing, Isolation and Characterization 

The long bones, femurs, and tibiae were collected from the same pair of donor rabbits, in the ice-cold transport medium (DMEM). All the procedures were carried out in an ice bath. After surface disinfection with 70% ethanol, the bones were cut open on both sides. Ice-cold 1× PBS was used to flush the bone by a 16-gauge needle attached to 1.0 mL syringe. Several rounds of flushing with ice-cold 1× PBS removed marrow to form the bone cavities. The removed marrow was flushed into a 100 μm strainer to break the clumps and to get a homogenous cell suspension. The strained cells were suspended in 1× PBS and centrifuged for 10 min at 180× *g* at 4 °C. RBC lysis buffer (ab204733, Abcam, Cambridge, UK) was added to the pelleted cells, incubated at 37 °C for 10 min and washed with ice-cold 1× PBS. The isolation of bone marrow mononuclear cells was performed by density gradient the ficoll-paque method [27]. The collected bone marrow cells were carefully layered over 3 mL of ficoll-paque. The volume of the ficoll was adjusted to the yield of the bone marrow. The layered tubes were centrifuged at 800× *g* for 30 min at room temperature. A white cloudy layer rich in mononuclear cells containing the MSCs was removed carefully using a sterile Pasteur pipette by circular movement. The collected mononuclear fraction was then transferred into a new tube and washed twice with PBS at 180× *g* for 10 min at room temperature. The cells were resuspended in 10 mL of complete DMEM medium. The cells were plated in a 100 mm TC treated dish with 10 mL of the media and incubated for 6 hours. Nonadherent cells were removed while adherent MSCs were detached for further purification by magnetic beads coated with MSCs specific phenotypic markers CD29 and CD90 (RoboSep™-S, STEMCELL Technologies, Vancouver, Canada) as per standard instructions to ensure homogenous pure population of MSCs. The purified fraction was further subjected to flow cytometric enumeration and confirmation of purity by labeling one part of the cellular fraction with MSC-specific phenotypic markers (the specific protein observed only in the membrane of MSCs) CD29, CD73, CD90 and exclusion markers CD34, and HLA-DR. The presence of CD34 or HLA-DR cells in the purified fraction indicates contamination of the MSCs with hematopoietic stem cells [28]. 

### 2.5. Viability and Osteo-induction 

We stained the isolated cells with 7-Aminoactinomycin D (7-AAD) and enumerated for CD29, CD73, CD90, CD34, and HLA-DR by flow cytometry. The viability of the purified fraction was expressed as percent viable cells. The cells were then subjected to osteoinduction by culturing 2 × 10^6^ cells per 30 mm TC dishes, with osteoinductive media DMEM containing 10% fetal bovine serum (FBS), 50 nm dexamethasone, 10 mM β-glycerophosphate, 0.2 mM L-ascorbic acid 2-phosphate and 100 units/mL penicillin-streptomycin for three weeks [29]. 

### 2.6. Osteoinduction Screening

The cells from osteoinductive media were washed with PBS and fixed with 4% paraformaldehyde solution in distilled water for 10 min at room temperature. The fixed cells were then stained with Fast Blue RR Salt, (Catalog No. FBS25-10 CAP (Sigma)), Naphthol AS-MX Phosphate Alkaline Solution (Sigma-Aldrich, Cat # 85L-2) mixture for ±20 min at room temperature. The excess stains were removed with successive washing with de-ionized water and documented for osteogenic induction through microscopic monitoring [30]. 

### 2.7. Surgical Procedure

**Figure 1:**
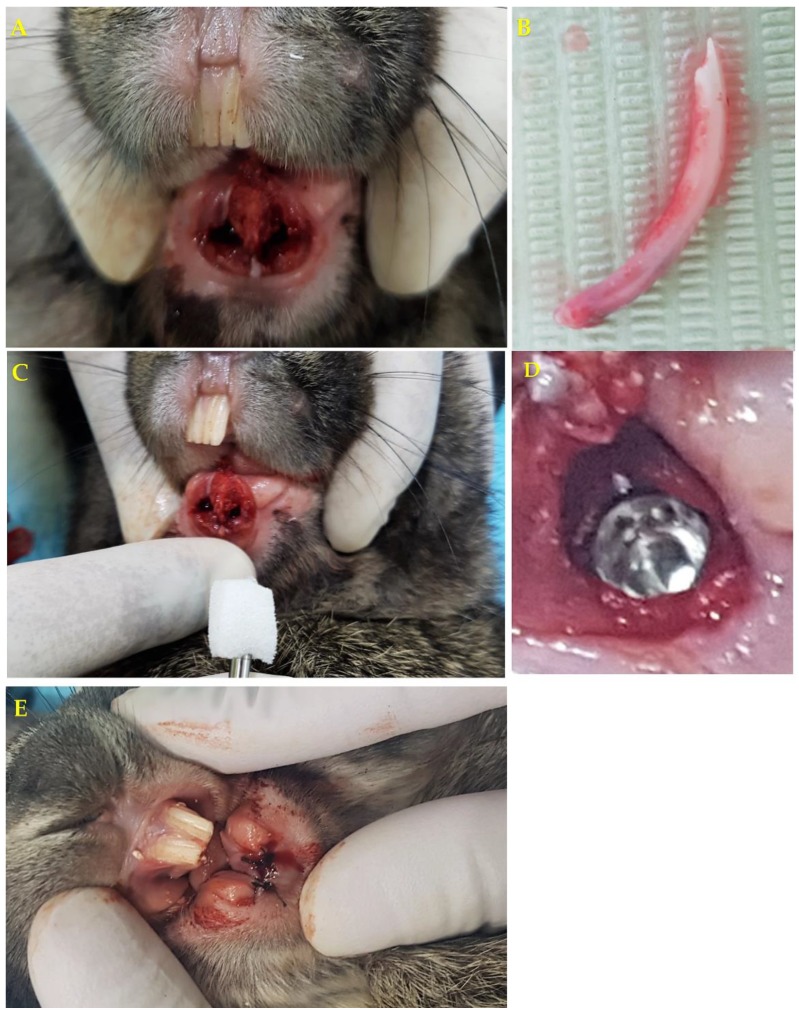
Surgical procedure illustrated. (**A**,**B**) The lower anterior teeth were carefully extracted. (**C**) Collagen sponges were inserted into sockets. (**D**) An implant was inserted into the socket through the channel in the sponge. (**E**) The sockets were closely sutured, and the upper anterior teeth were trimmed by 2 mm.

An equivalent surgical procedure was performed on rabbits from group C (*n* = 7/10) and group D (*n* = 7/10). Bilateral Mandibular incisors were carefully extracted, and the sockets were plugged with two serially placed cylindrical collagen sponges of 4 × 20 mm dimensions. (Gingistat, GABA VEBAS, Rome, Italy). Using a 2 mm wide, curved plugger, a channel was pierced along the center of the plug. The channel was expanded by gentle lateral compaction. The lateral compaction of the sponge allowed the implant insertion without pushing the collagen sponge apically. A mixture of 0.5 mL of autologous PRP containing osteoinductive BMMSCs (5 × 106 cells/mL) was injected into the collagen sponge of the right socket (subgroups CA and DA) [31]. The collagen sponge in the left socket was injected with plain PRP alone (subgroups CB and DB). Commercially available dental implants of 2.6 mm diameter and 10 mm length, (VITANE MaxiCare Implant System, Vitane Implant International, Strasbourg, France) were inserted into the collagen sponge of the incisor sockets, which was previously channeled by a plugger. The commercial dental implant had the entire surface Sandblasted and acid-etched (SLA treatment) till bone level; had a cylindrical-conical-cylindrical design; the neck was micro-threaded, and the apical area had vertical grooves and spherical apex. There was no significant torque application during insertion and the implant was intended to have frictional retention from the collagen sponge. The extraction socket was sutured closely (Figure 1).

The maxillary incisal edges were reduced by 2 mm to prevent injury to the implant site. We operated the diabetic animals four months post induction following a report that the duration of diabetes can significantly predispose to implant failures [32]. Appropriate medications were given to the operated rabbits. (Table S1 of supplementary material) 

### 2.8. Unoperated Animals

Three animals each from groups C and D (subgroups CU (*n* = 3/10) and DU (*n* = 3/10)) were left unoperated for backup. The unoperated diabetic and control animals were sacrificed at the end of the experiment to determine the baseline bone parameters around the mandibular incisor, and comparison of bone morphometry between the left and right side. Further, this paired comparison was used for statistical coherence.

### 2.9. Animal Euthanasia and Specimen Preparation 

No adverse events occurred during the experiment. We euthanized all the animals, including the unoperated rabbits (*n* = 20) six weeks post surgery, to evaluate early osseointegration. Euthanasia was performed by deep intramuscular infiltration of a triple dose of the anesthetic mixture containing 100 mg ketamine (Tekam^®^10 Hikma Farmaceutica. Portugal ) and 10 mg xylazine (Rompun^®^, Bayer Healthcare, LLC, Kansas, USA) as described elsewhere [33]. 

The mandible was dissected, and the two halves were separated at the symphysis. The right mandible of the operated animals of group C and D had an implant with stem cell therapy while the left mandible had implant without stem cell therapy. The mandibles of unoperated animals of group C and D had teeth on both sides. The specimens were fixed in 4% neutral formaldehyde and embedded in acrylic resin (Translucent Triad^®^ TruTray™, Dentsply Prosthetics, York, PA, USA).

### 2.10. μCT Scanning 

Thus, obtained enbloc bone specimens were scanned by a μCT scanner (Skyscan 1172, Bruker μCT, Kontich, Belgium) at the following specifications: power: 100 kV, 100 μA; integration time: 4730 ms; resolution: medium pixel 2 k. The beam hardening was reduced by using hardware filters (0.5 mm copper + 0.5 mm aluminum) and reconstruction program (NRecon, Bruker μCT, Kontich, Belgium) with slice setting of smoothing = 10, ring artifact correction = 7, and beam hardening correction = 9.

### 2.11. The Micromorphometric Analysis

**Figure 2:**
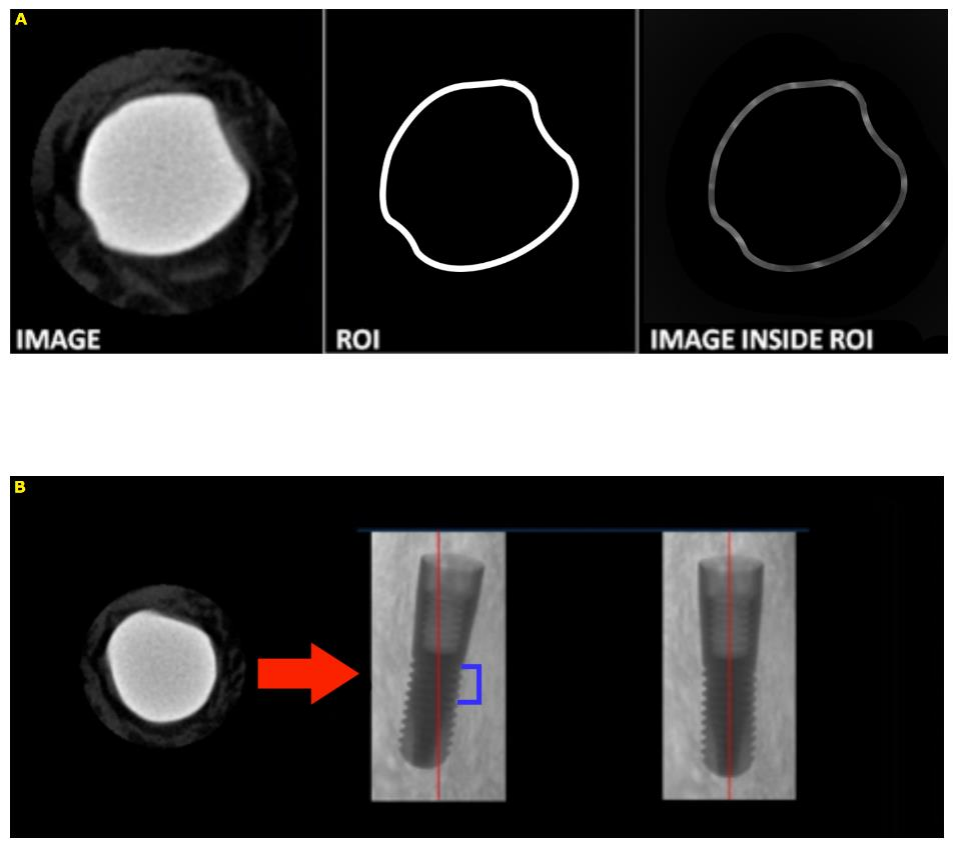
Selection method for the region of interest (ROI) and volume of interest (VOI). (**A**) ROI selection: The implant was selected based on its threshold level and this region was circumferentially expanded, creating a 0.75 mm zone around the implant. (**B**) Locating the implant axis in three planes: the X-ray beam (red arrow) was angulated perpendicular to the long axis of the implant. The blue line represents the area to be measured along the long axis of the implant.

The X-ray microtomographic images were analyzed using CT-Analyzer software (CT Analyzer version 1.11.10.0+) with direct 3D, based on a surface rendered volume model. The lower and upper grayscale of the analyzer was set at 19 and 160 respectively. The pixel size was fixed at 6.72 μm. The 3D analysis was started by demarcating the implant based on its threshold, and a region of interest (ROI) was selected 0.75 mm radially from the periphery of the implant (Figure 2A). The annular area along the 4 mm length of the implant starting from the first implant thread was selected as a volume of interest (VOI) (Figure 2B). The nomenclatures for the CT-analysis parameters are presented according to the guidelines for the assessment of bone microstructure using μCT by the American Society for Bone and Mineral Research (ASBMR) [34] (supplementary material Table S2).

### 2.12. Statistical Analysis 

We analyzed the data using IBM Statistical Package for the Social Sciences (SPSS) version 20 (SPSS Inc., Chicago, IL, USA). Descriptive analyses with histogram and boxplots were performed for all subgroups. 

Unoperated animals: The μCT bone parameters around right mandibular incisors were paired against that of the left side of each unoperated animal (subgroups CU and DU) to conduct “paired T-test.” An independent T-test was performed to determine the baselines of bone parameters around the mandibular incisors in subgroups CU and DU. 

Operated animals: The H0 was tested by the “paired T-test” of the μCT parameters of right implant specimen (subgroups CA and DA) paired against the left implant specimen (subgroups CB and DB) of each animal.

The research methods of the current study and manuscript presentation are compliant with ARRIVE guidelines. 

## 3. Results

### 3.1. Stem Cell Processing 

**Figure 3:**
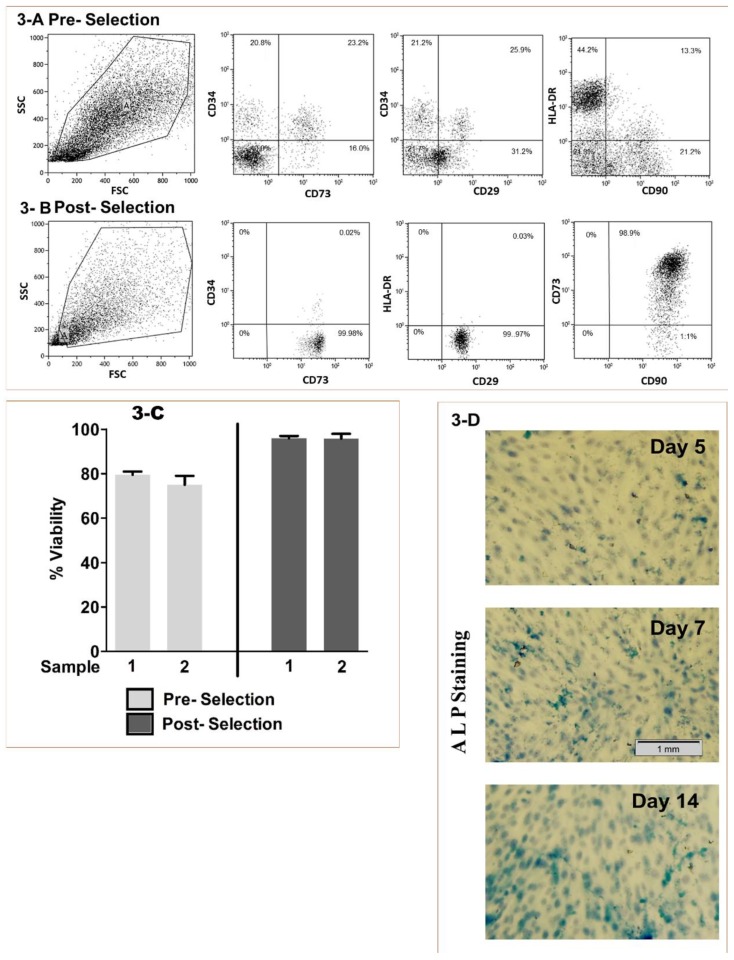
The purity of BMMSCs pre and post magnetic selection. (**A**) Preselection sample showing the presence of hematopoietic stem cells, MSCs, and other mononuclear cells. (**B**) Postselection samples showing a pure population of cells positive to CD29, CD73, and CD90 and negative to hematopoietic markers, CD34, and HLA-DR. (**C**) Viability of pre- and postselected fractions. (**D**) Alkaline phosphatase staining (ALP) showing osteogenic induction levels at 5, 7, and 14 days after induction.

The BMMSCs’ isolation and characterization resulted in ≥98% pure cells positive for CD 90, CD73, and CD29, but negative for CD34 and HLA-DR (Figure 3A,B). The viability of post magnetic bead purification of the BMMSCs resulted in ≥97% pure cells compared to the preselection of the bone marrow aspirate (Figure 3C). The predetermination of the BMMSCs by Alkaline phosphatase staining showed a gradual increase in osteocyte differentiation with time (Figure 3D). The above results of the preclinical quality assessment confirmed the cellular commitment under selection to differentiate into osteocytes.

### 3.2. Unoperated Animals (Subgroup CU and DU)

Paired T-test of the parameters of unoperated animals imply that there is no statistically significant difference in bone parameters surrounding right and left mandibular incisors in subgroups CU and DU (Table 1). The above results justify the “paired t-test” to analyze the implant specimens of the right side (subgroups CA and DA) against that of the left side (subgroups CB and DB) in the same animal.

The mean values of the bone volume fraction (BV/TV) and the trabecular thickness (TbTh) of the DU subgroup specimens are marginally lower than that of the CU group. However, the independent sample’s t-test suggests no statistically significant difference (Table 2). 

### 3.3. Operated Animals

**Figure 4:**
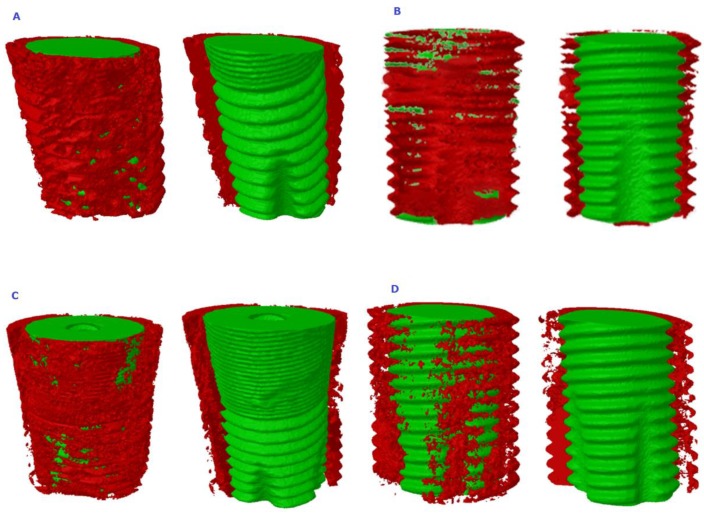
3D color-coded images of bone and implant; (**A**) specimen from a normal rabbit with stem cell therapy (subgroup CA); (**B**) specimen from a normal rabbit without stem cell therapy (subgroup CB); (**C**) specimen from a diabetic rabbit with stem cell therapy (subgroup DA); (**D**) specimen from a diabetic rabbit without stem cell therapy (subgroup DB).

Diabetic group: The implant specimen with stem cell therapy (subgroup DA) displayed statistically significant enhancement of osseointegration then implant specimens without stem cell therapy (subgroup DB) (Table 1, Figure 4).

Control group: The implant specimen with stem cell therapy (subgroup CA) displayed statistically significant enhancement of osseointegration than implant specimens without stem cell therapy (subgroup CB) (Table 1, Figure 4).

## 4. Discussion

There have been extensive studies on the enhancement of osseointegration through modification in physical characteristics of implants, surface coating, and stem cell therapy [35,36,37]. However, most of the studies were on healthy or osteoporotic subjects. Implants in diabetic patients are susceptible to diminished osseointegration and early implant failures [2]. Though reports suggest methods to enhance osseointegration in diabetic conditions [22,23,24], there were limited studies on the use of stem and progenitors from various anatomical niches for effective osseointegration. In the present study, we found that there was a significant increase in the bone quality around the implants under the influence or adjunct effect of osseo-inductive BMMSCs + PRP in both uncontrolled diabetic and normal rabbits. (Table 1) 

In vitro studies have reported the ability of BMMSCs to functionally differentiate into osteoblast with typical positivity for alkaline phosphatase, osteopontin, bone sialoprotein, osteocalcin, type I and III collagen and vascularization through various stages [38,39]. Few animal studies showed a successful differentiation of MSCs at the site of injury [40,41], but without any escalation to clinical trials. The initial results of our study concerning the viability and differentiation potential of BMMSCs in vitro were comparable with the published studies [31,42].

One earlier study suggested that the implant draped by osteoinductive MSC sheets enhanced osseointegration in type 2 diabetic rodents [43]. Further, the chitosan-hydroxyapatite composite coating on implant surface enhancing osseointegration in diabetic sheep was one of the studies done on higher vertebrate, which corroborated well with our observations [44]. It is well known that diabetes-induced hyperglycemia can diminish the osteoinductive ability of BMMSCs [45]. Increased levels of tissue necrotic factor (TNF) and reduction in bone morphogenetic protein-6 (BMP-6) are some of the reported mechanisms that could be implicated for delayed or failed osseointegration in diabetic cases [45,46]. Coating of polylactic-co-glycolic acid (PGLA) containing miR204 inhibitors and gold nanoparticles on the implant to overcome such attenuation has been reported to enhance implant homing [47]. However, the current study implies that the localized administration of inductive BMMSCs from a healthy rabbit can enhance osseointegration in diabetic conditions. Further studies on the impaired function of BMMSCs derived from diabetic animals are required to delineate the possibility of classical inflammatory microenvironment or loss of vasculature [48,49]. Additionally, the role of collagen, PRP, and BMMSCs at their capacity and various combinations need further evaluation.

Rabbits are the preferred research models for bone and implant-related studies [50]. While the metaphysis of long bones such as tibia is a common location for bone studies, high variation in the micromorphometric parameters of bone significantly affects the accuracy of the results [51]. Reports indicate a change in the location of bone by mm^3^ may alter the bone volume (BV/TV) by ±27% [52]. The body of the rabbit’s mandible varies from that of humans by having a larger amount of spongy bone and adipose tissue in the medullary spaces. However, the bone in the large diastema (±20 mm) between the incisors and premolars occupied by the incisor root is different. This part of the mandible has more bone volume and contains denser trabecular bone [53]. Due to its dimensions that (>100 mm^3^) can accommodate dental implants (up to 3 mm diameter and 12 mm length), facilitate performing surgical procedures, and enable postoperative evaluation, rabbit mandible enabled us to use a full-size commercially available dental implant and facilitated μCT analysis over a relatively larger volume of bone. A similar study in rodents lacks such advantages; however, the size of the rodents enables multiple in vivo μCT scans to study osseointegration at various time frames and, thereby, can determine the speed of osseointegration. The current study evaluated an early stage of osteointegration at six weeks post surgery. Further studies are required to evaluate the osseointegration at various time frames starting from four weeks to 12 weeks. There are no studies in the past, which could be used as a reference for studying the bone morphometry post stem cell coated bone formation in rabbit mandible. Therefore, our approach becomes novel, while it needs further investigations to appraise its clinical significance.

Though there are 38 bone parameters in μCT analysis, three parameters relevant to the study of implant osseointegration (BV/TV, TbTh, and bone implant contact (BIC)) were included in the study. It is evident from the literature that, BV/TV along with heterogeneous, anisotropic and asymmetric trabecular orientation are strong predictors of the bone mechanical properties [54,55]. Further, mean BV/TV and Tb Th of unoperated animals in the current study are within the range of values seen in the posterior region of medial femur and tibia, and cranium of the rabbits [21,22,23,24,25,26,27,28,29,30,31,32,33,34,35,36,37,38,39,40,41,42,43,44,45,46,47,48,49,50,51,52,53,54,55,56]. It may be noted that we did not use histological methods to evaluate bone formation; instead, we used micro CT measured 3D values of the whole area while being faster and nondestructive to the tissue. Moreover, the results of μCT analysis are comparable to that of histomorphometric analysis [21]. The μCT scanner used in our study (Skyscan 1172, Bruker μCT, Kontich, Belgium) used a round scanning technique to produce the BIC values ranging from 56 to 68%. In comparison, a spiral Nano-CT scanner (Skyscan 1275, Bruker μCT, Kontich, Belgium) could have produced images with lesser artifacts and values 2%–4% lower than round scan [57]. The BIC measured by different techniques may produce different values. Though the results obtained in the current experiment may not determine absolute values, the difference in the mean values between the groups carries significance.

An animal model can approximate the human’s physiology and biomechanics; nevertheless, differences in factors such as immunogenicity, turnover of bone, bone micromorphometry, availability of MSCs in the bone should be considered before extrapolating the results to the human application. The bone turnover, in particular, is significantly faster in the rabbits in comparison to humans. The implant placement in the current study simulates the clinical scenario of immediate implant placement in an extraction socket; we usually have space between the implant and the socket wall along most of the implant length. The collagen sponge, in addition to packing the space between the implant and socket walls, also facilitated a matrix for the BMMSCs. However, we do not know whether a conventional implant socket preparation without space between the implant and socket walls with stem cells would yield similar or better results, and this needs to be further investigated. Positive results of our current study may be escalated to a clinical trial on biomaterial scaffolds containing osseo-inducted autologous BMMSCs and PRP mixture in the future. In addition, direct use of “bone marrow aspirate concentrates” as a cellular graft will reduce the waiting time, add convenience, and can enable the clinicians to practice regenerative dentistry regularly.

## 5. Conclusions

From our current study, we observed that BMMSCs could be an ideal source for enhancement of implant osseointegration, especially in individuals with uncontrolled diabetes. The results of this study have further opened new avenues and possibilities for the use of MSCs from other minimal invasive sources like liposuction material or umbilical cord blood. The study results from the μCT scan and analysis document improved osseointegration of the implants in the diabetic rabbits. Therefore, it is evident that osseo-inducted MSCs play a major role in tissue repair and regeneration. Further insightful studies will be required in humans to ascertain the benefits of stem cell therapy. 

## Figures and Tables

**Table 1:** Results of paired t-tests and descriptive statistics for μCT parameters of operated subgroups and unoperated group.

Group/Subgroups	Parameters	Right Side		Left Side		*n*	95% CI for Mean Difference		*r*	*t*	p*	Df
		M	SD	M	SD		L	U				
Unoperated (CU + DU)	BV/TV	33.31	4.45	32.98	3.74	6	−1.06	1.72	0.962	0.605	0.57	5
	TbTh	419.51	96.82	402.98	70.49	6	−38.46	71.52	0.850	0.773	0.48	5
Control (Rt side = CA) (Lt side = CB)	BV/TV	35.68	5.81	32.09	5.37	7	2.92	4.26	0.995	13.17	0.00	6
	TbTh	436.83	101.57	382.05	86.26	7	24.15	85.41	0.951	4.38	0.01	6
	BIC	68.12	6.20	59.80	6.18	7	6.34	10.29	0.941	10.31	0.00	6
Diabetic (Rt side = DA) (Lt side = DB)	BV/TV	31.65	4.25	28.89	4.16	7	1.70	3.82	0.963	6.38	0.00	6
	TbTh	371.88	71.88	340.44	63.55	7	8.79	54.09	0.942	3.40	0.02	6
	BIC	66.68	5.42	56.75	7.15	7	3.99	15.87	0.506	4.09	0.01	6

CU: unoperated control group; DU: unoperated diabetic group; CA (Rt. Side): control group with stem cell therapy; CB (Lt. side): control group without stem cell therapy; DA (Rt. Side): diabetic group with stem cell therapy; DB (Lt. side): diabetic group without stem cell therapy; BV/TV: bone volume fraction (%); TbTh: trabecular thickness (μm); BIC: bone implant contact (%); *α = 0.05.

**Table 2:** Results of t-tests and descriptive statistics for μCT parameters of unoperated, individual subgroups.

Parameters	Groups						95% CI for Mean Difference		*t*	p*	df
	CU			DU							
	M	SD	*n*	M	SD	*n*	L	U			
BV/TV	34.15	3.85	6	32.31	4.10	6	−3.28	6.95	0.798	0.443	10
TbTh	430.54	100.94	6	391.95	58.50	6	−67.53	144.71	0.810	0.437	10

CU: unoperated control group; DU: unoperated diabetic group; BV/TV: bone volume fraction (%); TbTh: trabecular thickness (μm); *α = 0.05.

## Supplementary Materials

The following are available online at https://www.mdpi.com/1010-660X/56/4/148/s1, Table S1: Overview of the medication used for animal care during various stages of the study; Table S2: Description of the μCT morphometric parameters used in the study.
