# Supplementary material for: Bone Marrow Mesenchymal Stromal Cells (BMMSCs) Augment Osteointegration of Dental Implants in Type 1 Diabetic Rabbits: An X-Ray Micro-Computed Tomographic Evaluation
Source: Medicina (Kaunas). 2020 Mar 25;56(4):148. doi: 10.3390/medicina56040148 (PMC7230266; doi:10.3390/medicina56040148)
Supplement: Supplementary file 1 [file medicina-56-00148-s001.pdf]

TABLE S1: Overview of the medication used for animal care during various stages of the study

Stage of the Experiment	Group	Medication (trade name)	Dose/ kg body weight	Route	Duration (days)
Induction of Diabetes	Diabetic groups	Alloxan monohydrate	(100 mg/kg) in 5ml normal saline	i.v	q.d -1D
		5% Dextrose Normal saline	20 ml	S.C	S.O.S- 7D
		Human insulin (HuNil ®U40)		S.C	S.O.S- 7D
Pre-operative	Diabetic groups	Human insulin (HuNil ®U40)	1.5iu	S.C	q.d -1D
	All	cephalexin (Cephacare)	20mg	I.M	q.d -1D
		Buprenorphine (Vetergesic)	0.05 mg	S.C	q.d -1D
Operative (Sedation and anaesthesia)	All	Ketamine (Tekam®10 10mg/ml)	30mg	I.M	S.O.S to extend the duration
		Xylazine (Rompun)	3mg	I.M	q.d -1D
		2% lignocaine with 1:80000 adrenaline.	0.5ml	Infil	S.O.S to extend the duration
Post-operative	All	5% Dextrose Normal saline	20 ml	S.C	t.i.d-3D
	Diabetic groups	Human insulin (HuNil ®U40)		S.C	S.O.S- 3D
		Buprenorphine (Vetergesic)	0.05 mg	S.C	b.i,d - 3D
Euthanasia	All	Ketamine (Tekam®10 10mg/ml)	100mg	I.M	Lethal dose.
		Xylazine (Rompun)	10mg		

i.u: international units
i.v: intravenous injection
q.d: once a day/ single dose
b.i,d: twice a day
t.i.d: trice a day
S.C: subcutaneous injection
I.M: Deep intra muscular
Infil: Local infiltration
S.O.S: as required/ if necessary

Table S2. Description of the μCT morphometric parameters used in the study

ASBMR parameters	Synonyms	Symbol	Units	Description
Bone volume fraction	Percent bone volume; Percent object volume	BV/TV	%	Relative volume of bone in the selected 3-D peri implant volume. (volume of interest)
Trabecular thickness	Structure thickness	Tb.Th	μm	Direct 3D measure of mean trabecular thickness
Bone implant contact		BIC	%	The fraction of the implant surface in intimate contact with bone.
